# Matrix Metalloproteinase-7 Associated with Congestive Heart Failure in Peritoneal Dialysis Patients: A Prospective Cohort Study

**DOI:** 10.1155/2023/5380764

**Published:** 2023-05-02

**Authors:** Zhiwen Xiao, Jianwei Tian, Fen Zhang, Xiaohong Zhong, Tingting Zhang, Zhixiu Yi, Yanhong Lin, Cong Yang, Dan Tang, Nirong Gong, Jun Ai

**Affiliations:** State Key Laboratory of Organ Failure Research, National Clinical Research Center of Kidney Disease, Guangdong Provincial Key Laboratory of Renal Failure Research, Guangzhou Regenerative Medicine and Health Guangdong Laboratory, Guangdong Provincial Clinical Research Center for Kidney Disease, Division of Nephrology, Nanfang Hospital, Southern Medical University, 510005 Guangzhou, China

## Abstract

**Background:**

Matrix metalloproteinase-7 (MMP7) is markedly expressed in patients with chronic kidney disease; its expression in dialysate and role in patients undergoing peritoneal dialysis (PD) have not been well established.

**Methods:**

Participants undergoing PD from June 1st, 2015, to June 30th, 2020, were involved and were followed up every 3 months for the first year and every 6 months thereafter until death, PD withdrawal, or the end of the study. Data at each follow-up point were collected and analyzed for the association with congestive heart failure (CHF), PD withdrawal, and combined endpoint.

**Results:**

A total of 283 participants were included in this study. During a median follow-up of 21 months, 20 (7%) participants died, 93 (33%) withdrew from PD, and 105 (37%) developed CHF. A significantly increased level of serum and dialysate MMP7 was observed at baseline. Dialysate MMP7 presented a good linearity with serum MMP7. Baseline serum and dialysate MMP7 levels were associated with CHF in multivariable Cox proportional hazards regression models. After categorization, participants with high baseline MMP7 levels had a higher incidence of CHF (42%), and the hazard ratios (95% confidence intervals) were 1.595 (1.023-2.488). Interestingly, participants with higher serum MMP7 levels were trended to use dialysate with higher glucose concentration. However, the ultrafiltration volumes were not significantly increased. Higher MMP7 levels were also positively associated with PD withdrawal and combined endpoint.

**Conclusions:**

The expression of MMP7 in serum and dialysate was markedly increased and was tightly associated with the risk of CHF in PD patients. This finding suggests that the measurement of MMP7 may inform strategies for managing CHF at an earlier stage.

## 1. Introduction

With technique development and government medical insurance coverage, more patients with end-stage renal disease (ESRD) have received renal replacement therapy in China, during which more than 100,000 patients have received peritoneal dialysis (PD) [1]. Congestive heart failure (CHF), mainly caused by water retention, remains a common clinical challenge in PD patients, especially in those accompanied by anuria and/or ultrafiltration failure (UFF) [2]. The development of CHF in PD patients will increase the burden for both public health and the economy and lead to a higher frequency of readmission as well as poorer clinical outcomes including cardiovascular events and overall mortality [3–6]. Except for increasing glucose concentration of dialysate and transferring continuous ambulatory PD (CAPD) to intermittent PD (IPD) or to automatic PD (APD) [7–9], there are only a few effective methods to improve CHF. What is more, either exposure to higher glucose dialysate or increasing dialysis frequency might not improve CHF adequately, or some patients have to be transferred to hemodialysis (HD). In addition, irreversible damages had occurred before the clinical diagnosis has been established [10–12]. Early recognition and management of CHF in the PD population is an elephant in the room.

Matrix metalloproteinase-7 (MMP7), the smallest secreted MMP, plays an important role in many biological activities [13] and has been detected in the serum of patients with disease states [14–16]. MMP7 is significantly increased and acts as a noninvasive biomarker of prefibrotic signaling in patients with chronic kidney disease [17, 18]. Recently, Yin et al. found that MMP7 was highly expressed both in the serum and in the fluid discharged after dialysis in PD patients and could upregulate water channel protein—aquaporins 1 (AQP1), thereby enhancing water reabsorption of peritoneal mesothelial cell [19]. Thus, highly expressed MMP7 in the peritoneal dialysate might be associated with water transport across the peritoneal membrane and may be related to some clinical complications such as CHF. To confirm this, a prospective cohort study involving 283 participants was conducted in our department.

## 2. Subjects and Methods

### 2.1. Participants and Follow-Up

This was a single-center, prospective, cohort study. From June 1st, 2015 to June 30th, 2020, all participants who received peritoneal dialysis catheterization after diagnosis of chronic renal failure, with baseline clinical data and serum samples, aged 18 years or older and received PD treatment for more than 3 months, were eligible for this study. Participants who were reluctant to participate in the study or had irregular follow-up (less than once a year after dialysis or never after catheter implantation) were excluded. Follow-up was planned every 3 months for the first year and every 6 months thereafter until death, PD withdrawal, or June 30th, 2020. Healthy volunteers were also recruited and served as healthy control. All participants included in this study have provided written informed consent. The research protocol was approved by the Ethics Committee of Nanfang Hospital, Southern Medical University.

### 2.2. Data Collection and Calculation

Data were collected at the initial of PD, including demographic [age, sex, and body mass index (BMI)], primary disease of ESRD and comorbidity conditions (diabetes mellitus and hypertension), PD vintage, PD modality, and peritoneal equilibration test (PET) types at 1 month after PD. Serum hemoglobin (HGB), creatinine (Cr), albumin (ALB), calcium, phosphorus, intact parathyroid hormone (IPTH), dialysate glucose concentration (GLUC), 24 h urine volume, 24 h ultrafiltration (UF) volume, weekly total KT/V, systolic blood pressure, diastolic blood pressure, and left ventricular ejection fraction (LVEF) were collected at baseline (0 months, before PD) and also at 3, 6, 9, 12, 18, 24, 30, 36, 42, 48, 54, 60, and 66 months after PD. Antihypertensive drugs and phosphate binders were also recorded. An automatic biochemical analyzer (AU480; Olympus, Tokyo, Japan) was used to determine serum Cr, ALB, phosphorus, and calcium. A routine blood test analyzer (XN9000; Sysmex, Kobe, Japan) was used to measure HGB. Dialysate glucose concentration (%) = *Σ* (input volume × glucose concentration)/total input volume. Standard methods were performed to measure conventional weekly total KT/V and PET types [20].

### 2.3. Sample Collection and Measurement

Serum samples and 24 h peritoneal dialysate samples were obtained at baseline every three months during the first-year follow-up and annually thereafter. Blood samples were also collected from healthy volunteers. At the centrifuged condition of 4°C, 3000 r/min for 10 minutes, all supernatant liquids of samples which labeled with study identification numbers were preserved at −80°C. Samples were thawed at room temperature on the day of measurement and analyzed immediately. MMP7 was measured according to standard protocols in the central laboratory at baseline at the first-year follow-up and annually thereafter by ELISA kit (DY907, R&D Systems, Minneapolis, USA). Based on duplicate samples from study patients, the measured variation ranged from 3 to 8%.

### 2.4. Definitions and Outcomes

The primary outcome was an episode of CHF, defined as any clinically diagnosed CHF that required hospitalization. The first of these days was determined as the date of the incident. The diagnosis of CHF was based on the presence of characteristic symptoms and signs, and evidence of structural or functional abnormalities of the heart, according to the guidelines for the diagnosis and treatment of acute and chronic heart failure [21] (Supplementary Figure 1). An individual with definitive CHF would exhibit symptoms like shortness of breath or edema and receive medical treatment for CHF. As determined by two trained physicians, a definite diagnosis of CHF in our study requires that a minimum of one major and two minor or two major criteria be present concurrently [22], as shown in Supplementary Table 1. The secondary outcomes were PD withdrawal (including transfer to hemodialysis, renal transplantation, or death) and combined endpoint (including CHF, other cardiovascular diseases, stroke, or death), mainly based on the clinical diagnosis of the physician. Urine volume less than 100 ml in 24 hours was defined as anuria. UFF is defined as failure to achieve at least 400 ml of net UF during a 4 h dwell using 4.25% glucose.

### 2.5. Statistical Analyses

Dialysate MMP7 values were naturally log (ln) transformed to normalize the distribution. Continuous variables are shown as means and standard deviations (SD), and categorical variables were summarized as frequency and percentages. Baseline characteristics were compared between the two groups using *t*-test for continuous variables and the chi-square test for categorical variables. The random effects model was used to analyze the overall comparison of dialysate glucose concentration and UF volume over time between groups. The association between serum and dialysate MMP7 and CHF was assessed by using unadjusted and adjusted (age, sex, BMI, baseline LVEF, baseline UF, baseline total KT/V, loss of renal residual function, and mean dialysate glucose concentration and serum ALB of the first 12 months after dialysis) Cox regression analyses. We next categorized participants into two groups according to baseline serum MMP7 levels (MMP7 ≤ 4.80 ng/ml, MMP7 > 4.80 ng/ml). The Kaplan-Meier and Cox regression analyses were performed on the created variables. Cox proportional hazards models were also used to analyze the association of serum and dialysate MMP7 levels and PD withdrawal or combined endpoint. The unadjusted and adjusted hazard ratios (HR) were calculated with 95% confidence intervals (95% CI). All analyses were performed with Stata version 15 (Stata Statistical Software: Release 15). A two-sided *p* value < 0.05 was considered as statistically significant.

## 3. Results

### 3.1. Cohort Description

**Figure 1:**
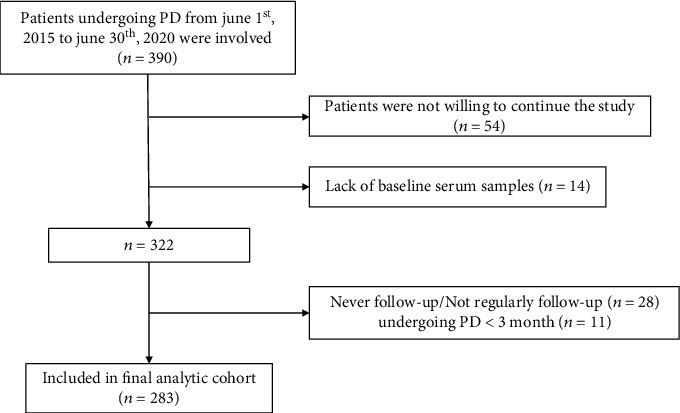
Flow chart of inclusion and exclusion. PD: peritoneal dialysis.

A total of 390 participants with ESRD who underwent PD in our department from June 1st, 2015 to June 30th, 2020 were enrolled. Among them, 107 were excluded: 54 were reluctant to continue the study, 14 were without baseline serum samples, 28 were without adequate follow-up, and 11 with PD duration of less than 3 months (Figure 1). A total of 283 participants were analyzed at last, and the mean age was 41.4 ± 13.5 years, 164 (58%) participants were male. During a median follow-up of 21 (11-37) months, 20 (7%) participants died, 93 (33%) withdrew from PD, and 105 (37%) developed CHF. Participants with CHF had lower baseline albumin, lower LVEF, and longer PD vintage. The details are shown in Table 1.

### 3.2. Expression of Serum and Dialysate MMP7

**Figure 2:**
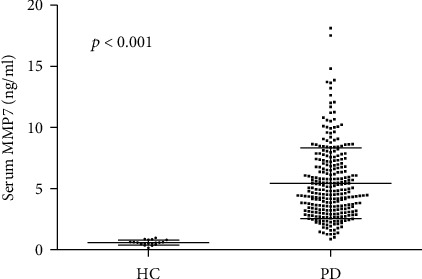
Expression of MMP7 in serum of peritoneal dialysis participants and healthy people. A significantly increased level of MMP7 in serum was observed in PD participants at baseline compared with healthy control. MMP7: matrix metalloproteinase-7; HC: healthy control; PD: peritoneal dialysis.

**Figure 3:**
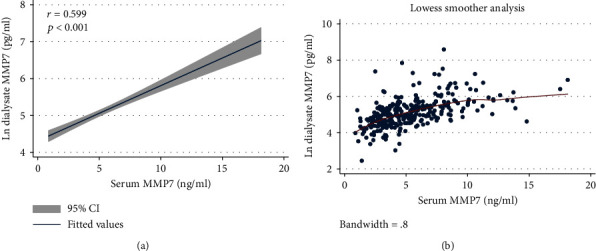
Correlation between serum and peritoneal dialysate MMP7. There was a significant linear correlation between peritoneal dialysate MMP7 and serum MMP7 (*r* = 0.599, *p* < 0.001). (a) Linear fitting curve. (b) Lowess smoother analysis. MMP7: matrix metalloproteinase-7; CI: confidence interval.

**Figure 4:**
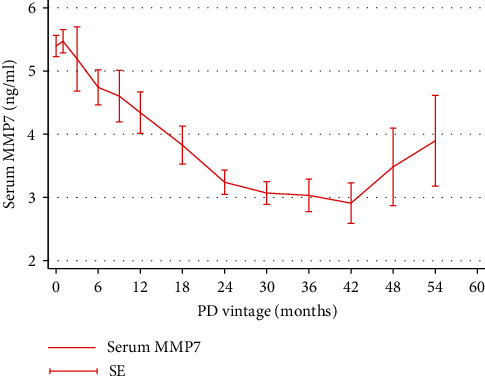
Expression of serum MMP7 changes over time in PD participants. The MMP7 levels rapidly decreased after dialysis. MMP7: matrix metalloproteinase-7; PD: peritoneal dialysis; SE: standard error.

As shown in Figure 2, serum MMP7 levels were significantly elevated at baseline in participants undergoing PD compared with healthy control (5.43 ± 2.91 vs. 0.59 ± 0.20 ng/ml, *p* < 0.001). Interestingly, dialysate MMP7 was also highly expressed (Table 1). Additionally, a favorable linear relationship was observed between serum MMP7 and dialysate MMP7 (*r* = 0.599, *p* < 0.001, Figure 3). After dialysis, serum MMP7 levels rapidly declined and then slowly elevated (Figure 4).

### 3.3. Association between MMP7 and CHF

**Figure 5:**
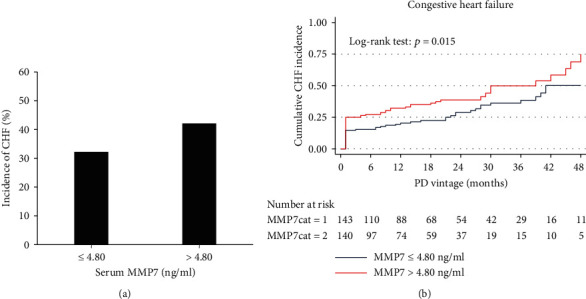
Association between MMP7 and CHF. Patients were categorized into two groups according to baseline serum MMP7 levels. MMP7cat = 1, MMP7 ≤ 4.80 ng/ml; MMP7cat = 2, MMP7 > 4.80 ng/ml. (a) Incidence of CHF in different groups. Patients with high MMP7 levels had a higher incidence of CHF (42%). (b) The Kaplan-Meier survival function curves showed a higher cumulative incidence of CHF in participants with increasing levels of baseline serum MMP7 (Log-rank test: *p* = 0.015). MMP7: matrix metalloproteinase-7; PD: peritoneal dialysis; CHF: congestive heart failure.

The association between MMP7 and CHF was analyzed at each follow-up time point. We found that only baseline serum (HR 1.089, 95% CI 1.024-1.159, *p* = 0.006) and dialysate (HR 1.558, 95% CI 1.212-2.002, *p* = 0.001) MMP7 levels were associated with CHF (Table 2). After adjusted by demographics (age, sex, and BMI), loss of renal residual function, baseline LVEF, UF volume, total KT/V, mean dialysate glucose concentration and serum ALB of the first 12 months after dialysis, baseline serum, and dialysate MMP7 levels were still significantly associated with CHF (Table 2). MMP7 levels at other follow-up points were not associated with CHF (Supplementary Table 2). As shown in Figures 5(a) and 5(b), participants in the group with the high baseline serum MMP7 levels (>4.80 ng/ml) had a higher incidence of CHF (42%), and the risk of CHF greatly increased with the HR of 1.577 (95% CI 1.067-2.331, *p* = 0.02) by unadjusted analysis and of 1.595 (95% CI 1.023-2.488, *p* = 0.04) after adjusted analysis, compared to the lower baseline serum MMP7 group (Table 3).

### 3.4. Relationship between MMP7 and Longitudinal Changes in Clinical Parameters of PD

**Figure 6:**
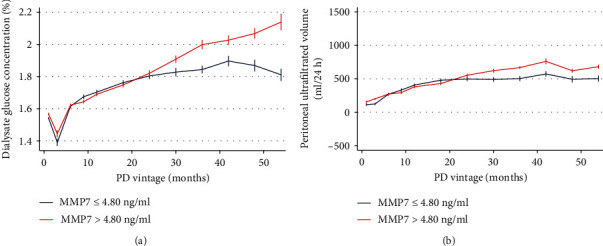
Longitudinal changes over time of dialysate glucose concentration and ultrafiltration volume. Participants were categorized into two groups according to baseline serum MMP7 levels (MMP7cat = 1, MMP7 ≤ 4.80 ng/ml; MMP7cat = 2, MMP7 > 4.80 ng/ml). (a) Participants with higher serum MMP7 levels were trended to be treated with higher dialysate glucose concentration. (b) The ultrafiltration volume appeared to be similar between groups with different MMP7 levels. MMP7: matrix metalloproteinase-7; PD: peritoneal dialysis; SE: standard error.

Since CHF might be tightly associated with water retention caused by UFF and decreased urine output [23], we next analyzed the relationships between MMP7 and longitudinal changes of UF volume as well as dialysate glucose concentration. Interestingly, participants with higher serum MMP7 levels trended to use dialysate with higher glucose concentrations earlier over time (Figure 6(a)), but the UF volume appeared to be similar between groups with different MMP7 levels (Figure 6(b)).

### 3.5. Association between MMP7 and Secondary Outcomes

**Figure 7:**
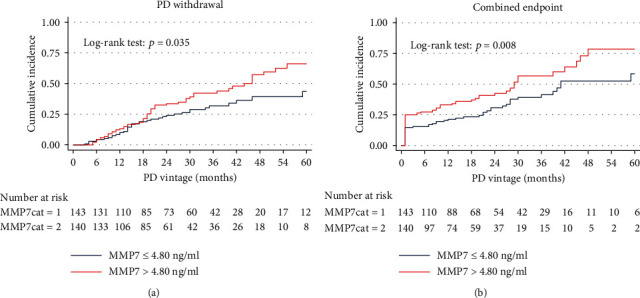
Kaplan-Meier curves for PD withdrawal and combined endpoint. Participants were categorized into two groups according to baseline serum MMP7 levels (MMP7cat = 1, MMP7 ≤ 4.80 ng/ml; MMP7cat = 2, MMP7 > 4.80 ng/ml). The Kaplan-Meier survival function curves showed a higher cumulative incidence of PD withdrawal (*p* = 0.035) and combined endpoint (*p* = 0.008) in participants with increasing levels of baseline serum MMP7. PD withdrawal was defined as death, transfer to hemodialysis, or renal transplantation. Combined endpoint was defined as death, CHF, cardiovascular diseases, or stroke. MMP7: matrix metalloproteinase-7; PD: peritoneal dialysis; HR: hazard ratio; CI: confidence interval; CHF: congestive heart failure.

During the follow-up period, 93 participants withdrew from PD, and 115 participants had reached a combined endpoint. The Kaplan-Meier survival function curves showed a higher cumulative incidence of PD withdrawal and combined endpoint in participants with increasing levels of baseline serum MMP7 (Figure 7). Serum MMP7 levels were associated with PD withdrawal (HR 1.090, 95% CI, 1.028-1.550, *p* = 0.004) and combined endpoint (HR, 1.083, 95% CI, 1.011-1.580, *p* = 0.02). A similar correlation was found also on the dialysate MMP7 levels. The results were consistent after adjustment for demographics (age and sex), the mean values of dialysate glucose concentration, urine volume, serum creatinine, serum phosphate, hemoglobin, and KT/V during the first 12 months after dialysis (Table 4).

## 4. Discussion

In the prospective cohort study, we demonstrated that MMP7 was highly expressed in the serum and dialysate of participants undergoing PD. The level of serum MMP7 first decreased and then gradually increased with the prolonged PD vintage. Interestingly, baseline serum and dialysate MMP7 levels were associated with CHF. Participants with high baseline serum MMP7 levels had an increased risk of developing CHF with an HR of 1.595 (95% CI 1.023-2.488, *p* = 0.04). Although participants with higher MMP7 levels trended to use higher glucose concentrations of dialysate, UF volume did not improve significantly. These results suggested that MMP7 might play an important role in PD patients.

Some studies have shown that the expression of MMP7 was almost undetectable in healthy people [24, 25]. However, its expression was significantly induced in serum and urine in both acute and chronic kidney injury models [26]. Fang et al. found that higher urine MMP7 levels were correlated with an increased risk of progressive acute kidney injury [27]. However, other studies confirmed that urine MMP7 levels were positively associated with renal fibrosis scores and negatively correlated with renal function progression [24, 28]. Peritoneal dialysate is incessantly stayed in the peritoneum and is exchanged 4 times a day, which is the crucial way for the clearance of solute and fluid in PD patients. In addition, high glucose exposure could promote the expression of MMP7 [29]. Therefore, we speculated that MMP7 might also be significantly expressed in the peritoneal dialysate. We did find the markedly expressed MMP7 in both serum and dialysate of PD patients. The quite well-linear association between MMP7 in serum and in dialysate suggests that dialysate MMP7 might be mainly excreted from serum.

Functionally, MMP7 could degrade a variety of extracellular matrix components, activate proteases, and play a key role in regulating diverse cellular processes such as matrix remodeling and epithelial-mesenchymal transformation (EMT) [14, 30]. However, the expression and role of MMP7 in the dialysate have not been well established. Our previous cross-sectional study had shown that dialysate MMP7 was negatively correlated with UF volume in PD patients [19]. The reduction of UF volume might be directly related to water retention, leading to the occurrence of CHF. We then focused on the association between MMP7 and CHF. Due to sufficient patient–doctor interactions in our cohort, participants were able to report their symptoms and signs of CHF to the physicians in charge timely and could be admitted to the hospital for early intervention. These might account for the seemingly high number of hospitalizations for CHF. Interestingly, both baseline serum and dialysate MMP7 did associate with CHF even in the fully adjusted model. When categorized into two groups, participants with high MMP7 levels had a higher risk of developing CHF, which was 1.595 times higher than that in the group with low MMP7 levels. These data indicated that the markedly increased baseline MMP7 might be a potential early marker for CHF.

Here, we did confirm that MMP7 was associated with CHF, but whether MMP7 is a causal factor of CHF is not sure. Interestingly, we found that the difference in UF volume between the MMP7 groups was similar after a longitudinal study over time, although participants in the high MMP7 group trended to use dialysate with a higher glucose concentration. Initially, due to possible differences in concentrations of serum and intraperitoneal MMP7, serum MMP7 might be excreted into the peritoneal cavity through the dialysate. Serum MMP7 decreased first with time due to the clearance of dialysate. However, the long-term continuous exposure of the peritoneum to high glucose dialysate might have contributed to the nonphysiological elevation of MMP7 in the dialysate. Serum MMP7 tended to increase with the duration of dialysis. In our previous studies, we have found that MMP7 might affect UF volume by altering the permeability of peritoneal mesothelial cells via the water channel protein—AQP1 and leading to cell enlargement. High MMP7 levels were associated with reduced UF volume. Due to the clinical presentation, the treatment for participants with decreased UF volume would be modified including the use of dialysate with higher glucose concentrations, since all decisions were made by the physicians. Nevertheless, its effect on improving UF in participants with high MMP7 was limiting, which might account for the high incidence of CHF events.

The etiology of CHF in PD patients is particularly complex. Atherosclerosis, hypertension, fluid overload, and inadequate dialysis prescription or poor adherence might be the key contributing factors [31–33]. Recurrence of CHF increases the readmission rate of PD patients, worsens the prognosis, increases the incidence of mortality and cardiovascular events, and aggravates medical and economic burdens [3–6, 33, 34]. In most patients, the diagnosis can only be established when overt manifestations of CHF are present. Irreversible cardiac damages have occurred before diagnosis [35, 36]. Therefore, earlier detection is an urgent clinical problem. In our study, baseline MMP7 levels were tightly associated with CHF, indicating that serum and dialysate MMP7 might act as one of the early biomarkers. For PD patients with higher MMP7 levels, we should pay more attention to the follow-up, especially to the balance of intake and outflow, blood pressure and weight change, clinical symptoms, etc. All in all, the noninvasive detection of MMP7 might help physicians make judgments and conduct clinical interventions earlier to prevent CHF.

The following strengths were shown in our study. Firstly, it was a prospective study in which the outcome of each participant was observed during the follow-up period. Secondly, the levels of MMP7 were measured in our central key laboratory with standard procedures which reduced measurement errors. Most importantly, this study has presented several characteristics of MMP7 in PD patients, including the high expression in dialysate, the tight association with the occurrence of CHF, and the potential early biomarker for CHF. However, several limitations should be considered when accounting for the findings. As our results were reflected in a single-center cohort, validation studies from other populations are needed. If MMP7 levels were measured at each CHF event, the results might be more convincing. Due to the small number of deaths during follow-up, the association between MMP7 and mortality was not explored. The research on the mechanism should be further strengthened.

## 5. Conclusion

This study provided a unique perspective on the expression of MMP7 in patients undergoing PD and identified its association with CHF. If further confirmed, measurement of serum and dialysate MMP7 might help clinicians detect CHF events at an earlier stage, conduct proper treatment strategies, and improve the clinical prognosis of PD patients.

## Figures and Tables

**Table 1:** Clinical characteristics of the study cohort.

General characteristics	Overall (*n* = 283)	Without CHF (*n* = 178)	With CHF (*n* = 105)	*p* ^a^
Age (y)	41.4 ± 13.5	41.9 ± 12.5	40.5 ± 15.1	0.39
Male, *n* (%)	164 (58)	107 (60)	57 (54)	0.34
MAP (mmHg)	113.3 ± 17.6	112.2 ± 16.4	115.2 ± 19.4	0.18
BMI (kg/m^2^)	22.2 ± 9.4	22.1 ± 11.4	22.3 ± 3.8	0.86
Primary renal diseases, *n* (%)				0.06
Chronic glomerulonephritis	204 (72)	135 (76)	69 (66)	
Diabetic nephropathy	39 (14)	19 (11)	20 (19)	
Hypertensive nephropathy	34 (12)	18 (10)	16 (15)	
Others	6 (2)	6 (3)	0 (0)	
Comorbidities, *n* (%)				
Diabetes mellitus	39 (14)	19 (11)	20 (19)	0.05
Hypertension	264 (93)	165 (93)	99 (94)	0.61
Baseline lab examinations				
Serum creatinine (*μ*mol/l)	1005.8 ± 393.4	1017.0 ± 354.9	987.1 ± 451.6	0.54
Serum albumin (g/l)	35.4 ± 5.6	36.2 ± 5.7	34.1 ± 5.2	0.002
Blood hemoglobin (g/l)	81.9 ± 17.7	83.0 ± 17.7	80.1 ± 17.5	0.19
Serum phosphate (mmol/l)	2.03 ± 0.67	2.03 ± 0.64	2.02 ± 0.73	0.86
Serum PTH (pg/ml)	344.3 ± 229.6	364.2 ± 254.9	311.9 ± 177.7	0.09
Serum MMP7 (ng/ml)	5.43 ± 2.91	5.14 ± 2.63	5.93 ± 3.28	0.03
Ln dialysate MMP7 (pg/ml)	5.11 ± 0.78	5.00 ± 0.78	5.30 ± 0.75	0.003
LVEF, %	64.6 ± 10.7	66.6 ± 8.6	61.3 ± 12.7	<0.001
Baseline PD characteristics				
PD modality, *n* (%)				
CAPD	265 (94)	164 (92)	101 (96)	0.18
Type of dialysates, *n* (%)				
Conventional	283 (100)	178 (100)	105 (100)	—
Dialysate GLUC, %	1.56 ± 0.15	1.55 ± 0.13	1.58 ± 0.16	0.08
UF volume (ml/24 h)	200 (-175-400)	200 (-200-450)	200 (-100-400)	0.68
Urine volume (L/24 h)	0.95 ± 0.54	0.94 ± 0.52	0.96 ± 0.58	0.74
KT/V score	2.44 ± 0.84	2.40 ± 0.69	2.50 ± 1.03	0.32
D/P creatinine ratio	0.68 ± 0.13	0.67 ± 0.13	0.69 ± 0.12	0.27
PD vintage, *m*	21 (11-37)	19 (11-33)	29 (12-44)	0.01
Medication, *n* (%)				
Antihypertensive drugs	262 (93)	162 (91)	100 (95)	0.19
Outcomes, *n* (%)				
Death	20 (7)	9 (5)	11 (11)	0.10
PD withdrawal	93 (33)	57 (32)	36 (34)	0.70

Continuous variables were expressed as mean ± SD or median (25th percentile-75th percentile). Categorical variables were expressed as *n* (%). MAP, BMI, Ln dialysate MMP7, KT/V, and PET were calculated by the formulas mentioned before. CHF: congestive heart failure; MAP: mean arterial pressure; BMI: body mass index; ESRD: end-stage renal disease; PTH: parathyroid hormone; MMP7: matrix metalloproteinase-7; LVEF: left ventricular ejection fraction; PD: peritoneal dialysis; CAPD: continuous ambulatory peritoneal dialysis; GLUC: glucose concentration; UF: ultrafiltration; PET: peritoneal equilibration test. ^a^*p* for comparisons between with and without CHF groups by *t*-test, Kruskal-Walli's test, or chi-squared test for continuous and categorical variables, respectively.

**Table 2:** Cox regression analysis for CHF.

Variables	Unadjusted		Adjusted^a^	
	HR (95% CI)	*p*	HR (95% CI)	*p*
Serum MMP7 (ng/ml)	1.089 (1.024-1.159)	0.006	1.093 (1.015-1.177)	0.02
Ln dialysate MMP7 (pg/ml)	1.558 (1.212-2.002)	0.001	1.567 (1.137-2.159)	0.006
Age (y)	0.986 (0.972-1.001)	0.08	0.997 (0.982-1.014)	0.77
Male	0.895 (0.609-1.314)	0.57	0.987 (0.576-1.691)	0.96
BMI (kg/m^2^)	1.003 (0.986-1.020)	0.73	0.993 (0.972-1.013)	0.49
Baseline LVEF, %	0.960 (0.941-0.980)	<0.001	0.966 (0.943-0.989)	0.004
Mean dialysate GLUC, %	2.669 (1.006-7.077)	0.050	2.384 (0.716-7.944)	0.16
Baseline UF (ml)	1.000 (0.999-1.000)	0.56	0.999 (0.999-1.000)	0.12
Mean serum ALB (g/l)	0.946 (0.907-0.987)	0.01	0.992 (0.942-1.044)	0.75
Loss of RRF	1.869 (1.259-2.776)	0.002	1.794 (1.123-2.864)	0.01
TKT/V	1.049 (0.822-1.338)	0.70	1.098 (0.758-1.590)	0.62

Ln dialysate MMP7, BMI, and TKT/V were calculated by the formulas mentioned before. Dialysate glucose concentration, urine volume, serum ALB, and TKT/V were the average values during the first 12 months after dialysis. CHF: congestive heart failure; MMP7: matrix metalloproteinase-7; BMI: body mass index; LVEF: left ventricular ejection fraction; GLUC: glucose concentration; UF: ultrafiltration; ALB: albumin; RRF: residual renal function; HR: hazard ratio; CI: confidence interval. ^a^Adjusted with age, sex, BMI, baseline LVEF, baseline UF, baseline total KT/V, loss of residual renal function, mean dialysate glucose concentration, and serum ALB of the first 12 months after dialysis.

**Table 3:** Cox regression analysis for CHF with categorized serum MMP7.

Serum MMP7 (ng/ml)	Unadjusted		Adjusted^a^	
	HR (95% CI)	*p*	HR (95% CI)	*p*
≤4.80	1 (refer)	—	1 (refer)	—
>4.80	1.577 (1.067-2.331)	0.02	1.595 (1.023-2.488)	0.04

Baseline serum MMP7 was categorized into two groups. BMI and KT/V were calculated by the formulas mentioned before. CHF: congestive heart failure; MMP7: matrix metalloproteinase-7; HR: hazard ratio; CI: confidence interval; BMI: body mass index; LVEF: left ventricular ejection fraction; UF: ultrafiltration; ALB: albumin. ^a^Adjusted with age, sex, BMI, baseline LVEF, baseline UF, baseline total KT/V, loss of residual renal function, mean dialysate glucose concentration, and serum ALB of the first 12 months after dialysis.

**Table 4:** Cox regression analysis for PD withdrawal and combined endpoint.

Variables	PD withdrawal		Combined endpoint	
	HR (95% CI)	*p* ^a^	HR (95% CI)	*p* ^b^
Serum MMP7, ng/ml	1.090 (1.028-1.550)	0.004	1.083 (1.011-1.580)	0.02
Ln dialysate MMP7, pg/ml	1.468 (1.152-1.870)	0.002	1.532 (1.131-2.076)	0.006

KT/V was calculated by the formulas mentioned before. PD: peritoneal dialysis; MMP7: matrix metalloproteinase-7; HR: hazard ratio; CI: confidence interval; DKD: diabetic kidney disease; ESRD: end-stage renal disease. ^a^Adjusted with demographics (age and sex), the mean values of dialysate glucose concentration, urine volume, serum creatinine, serum phosphate, hemoglobin, and KT/V during the first 12 months after dialysis. ^b^Adjusted with demographics (age and sex), DKD to ESRD, the mean values of dialysate glucose concentration, urine volume, serum albumin, serum phosphate, hemoglobin, and KT/V during the first 12 months after dialysis.

## Data Availability

The data used and analyzed in our research are available from the corresponding authors upon request.

## Supplementary Materials

Supplementary Materials Supplementary Table 1: definitions of congestive heart failure (CHF) by study cohort. Supplementary Table 2: Cox regression analysis of MMP7 levels and congestive heart failure in different follow-up periods. Supplementary Figure 1: diagnostic algorithm for a diagnosis of heart failure of nonacute onset.
