# Supplementary material for: Matrix Metalloproteinase-7 Associated with Congestive Heart Failure in Peritoneal Dialysis Patients: A Prospective Cohort Study
Source: Mediators Inflamm. 2023 May 2;2023:5380764. doi: 10.1155/2023/5380764 (PMC10169244; doi:10.1155/2023/5380764)
Supplement: Supplementary Materials — Supplementary Table 1: definitions of congestive heart failure (CHF) by study cohort. Supplementary Table 2: Cox regression analysis of MMP7 levels and congestive heart failure in different follow-up periods. Supplementary Figure 1: diagnostic algorithm for a diagnosis of heart failure of nonacute onset. [file 5380764.f1.docx]

**Contents for the supplemental material**

**Supplementary Tables：**

Supplementary Table 1. Definitions of Congestive Heart Failure (CHF) by Study Cohort

Supplementary Table 2. Cox Regression Analysis of MMP7 Levels and Congestive Heart Failure in Different Follow-up Periods

**Supplementary Figures:**

Supplementary Figure 1. Diagnostic Algorithm for A Diagnosis of Heart Failure of Non-acute Onset

**Supplementary Table 1. Definitions of Congestive Heart Failure (CHF) by Study Cohort**

| Major criteria | Minor criteria |
| --- | --- |
| Orthopnea | Dyspnea on ordinary exertion |
| Paroxysmal nocturnal dyspnea | Nocturnal cough |
| Hepatojugular reflux | Bilateral ankle edema |
| Jugular venous distension | Tachycardia |
| Pulmonary rales | Pleural effusion |
| Third heart sound | Hepatomegaly |
| Radiographic evidence of acute pulmonary edema or cardiomegaly | Radiographic evidence of pulmonary vascular engorgement |

A definite diagnosis of CHF requires that a minimum of one major and two minor or two major criteria be present concurrently, including physical examination signs and symptoms, diagnostic imaging tests, etc.

**Supplementary Table 2. Cox Regression Analysis of MMP7 Levels and Congestive Heart Failure in Different Follow-up Periods**

| **Variables** | **Unadjusted** | | **Adjusted ^a^** | | |
| --- | --- | --- | --- | --- | --- |
|  | **HR (95%CI)** | **p** | **HR (95%CI)** | | **p** |
| **Serum MMP7, ng/ml**  **0m** | 1.089 (1.024-1.159) | 0.006 | | 1.093 (1.015-1.177) | 0.02 |
| **6m** | 1.051 (0.958-1.151) | 0.29 | | 1.067 (0.939-1.213) | 0.32 |
| **12m** | 0.990 (0.866-1.131) | 0.88 | | 0.956 (0.817-1.118) | 0.58 |
| **24m** | 0.895 (0.637-1.256) | 0.52 | | 0.953 (0.596-1.525) | 0.84 |
| **36m** | 0.906 (0.648-1.267) | 0.57 | | 1.207 (0.736-1.982) | 0.47 |
| **Ln dialysate MMP7, pg/ml** |  |  | |  |  |
| **0m** | 1.558 (1.212-2.002) | 0.001 | | 1.567 (1.137-2.159) | 0.006 |
| **3m** | 1.184 (0.829-1.690) | 0.35 | | 1.133 (0.699-1.837) | 0.61 |
| **6m** | 1.455 (0.924-2.289) | 0.11 | | 0.879 (0.479-1.616) | 0.68 |
| **9m** | 1.621 (1.028-2.555) | 0.03 | | 1.225 (0.493-3.039) | 0.66 |
| **12m** | 1.690 (1.170-2.443) | 0.005 | | 1.624 (0.851-3.099) | 0.14 |

BMI, KT/V were calculated by formulas mentioned before. MMP7, matrix metalloproteinase 7. HR, hazard ratio. CI, confidence interval. BMI, body mass index. LVEF, left ventricular ejection fraction. UF, ultrafiltration. ALB, albumin.

a Adjusted with age sex, BMI, baseline LVEF, baseline UF, baseline total KT/V, loss of residual renal function, and mean dialysate glucose concentration and serum ALB of the first 12 months after dialysis.

**Supplementary Figures:**


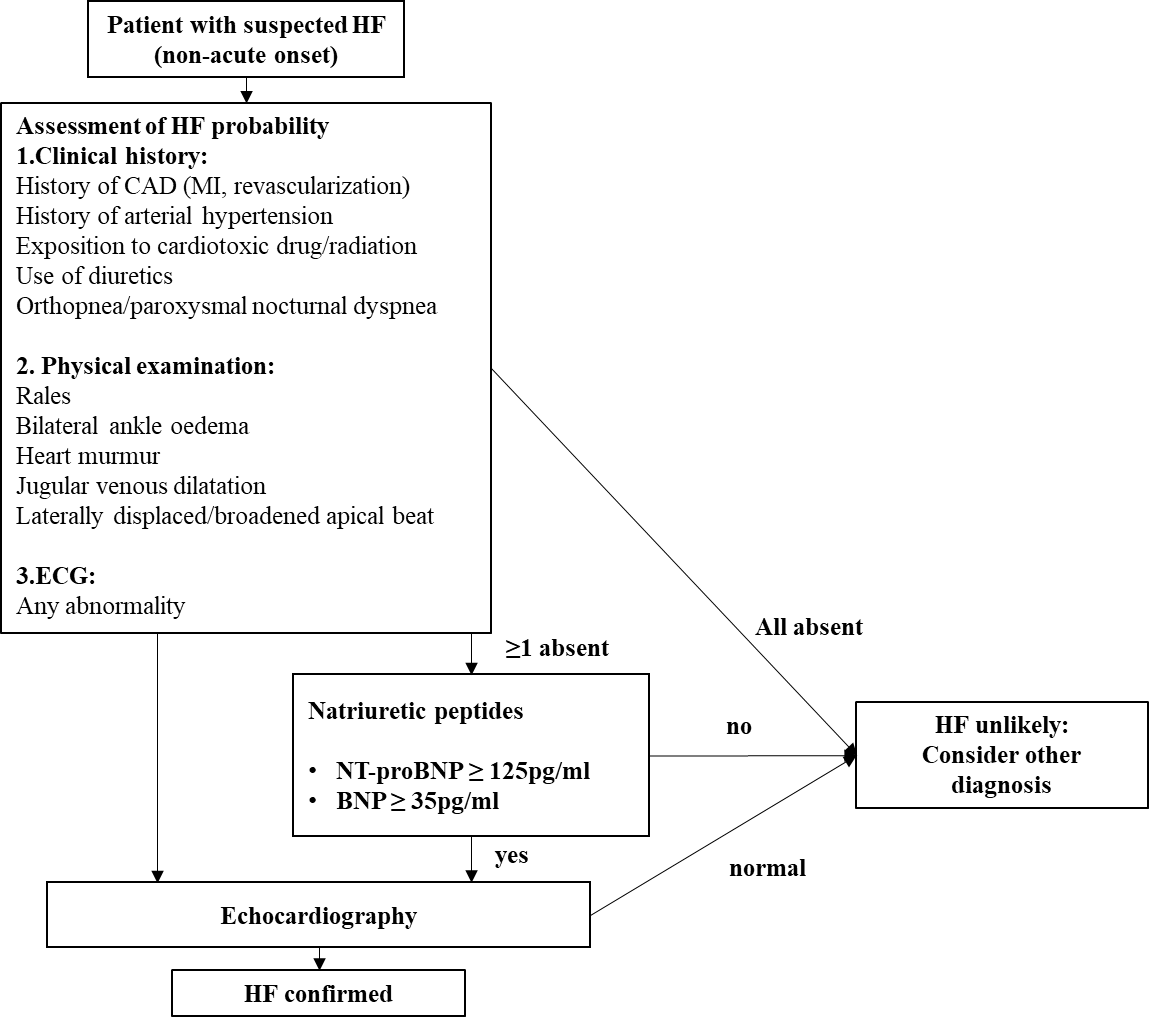


**Supplementary Figure 1. Diagnostic Algorithm for A Diagnosis of Heart Failure of Non-acute Onset**

Laboratory and imaging examinations are lack of specificity in the diagnosis of CHF in PD patients. There is no recognized cut-off value for traditional biomarkers, such as NT-proBNP, in the dialysis population at present, and its application is still controversial^[1-3]^. Thus, the diagnosis of CHF in our study was based on the presence of characteristic symptoms and signs, and evidence of structural or functional abnormalities of the heart.

Reference

[1] Haapio M, Honkanen E, Ronco C. Brain natriuretic peptide in peritoneal dialysis patients[J]. Contrib Nephrol,2009,163:110-116.

[2] Chung J H, Yun N R, Ahn C Y, et al. Relationship between Serum N-Terminal Pro-Brain Natriuretic Peptide Level and Left Ventricular Dysfunction and Extracellular Water in Continuous Ambulatory Peritoneal Dialysis Patients[J]. Electrolyte Blood Press,2008,6(1):15-21.

[3] Maisel A, Mueller C, Adams K J, et al. State of the art: using natriuretic peptide levels in clinical practice[J]. Eur J Heart Fail,2008,10(9):824-839.
